# Increased Adipogenesis in Cultured Embryonic Chondrocytes and in Adult Bone Marrow of Dominant Negative Erg Transgenic Mice

**DOI:** 10.1371/journal.pone.0048656

**Published:** 2012-11-14

**Authors:** Sébastien Flajollet, Tian V. Tian, Ludovic Huot, Nathalie Tomavo, Anne Flourens, Muriel Holder-Espinasse, Marion Le Jeune, Patrick Dumont, David Hot, Frédéric Mallein-Gerin, Martine Duterque-Coquillaud

**Affiliations:** 1 CNRS UMR 8161, Institut de Biologie de Lille, Université de Lille Nord de France, Institut Pasteur de Lille/IFR142, Lille, France; 2 Transcriptomics and Applied Genomics, Institut Pasteur de Lille – Center for Infection and Immunity of Lille, U1019, UMR 8204, Lille, France; 3 CNRS, FRE 3310 – Dysfonctionnement de l'Homéostasie Tissulaire et Ingénierie Thérapeutique, IBCP, Université Lyon 1, Univ Lyon, Lyon, France; University of Western Ontario, Canada

## Abstract

In monolayer culture, primary articular chondrocytes have an intrinsic tendency to lose their phenotype during expansion. The molecular events underlying this chondrocyte dedifferentiation are still largely unknown. Several transcription factors are important for chondrocyte differentiation. The Ets transcription factor family may be involved in skeletal development. One family member, the *Erg* gene, is mainly expressed during cartilage formation. To further investigate the potential role of Erg in the maintenance of the chondrocyte phenotype, we isolated and cultured chondrocytes from the rib cartilage of embryos of transgenic mice that express a dominant negative form of Erg (DN-Erg) during cartilage formation. DN-Erg expression in chondrocytes cultured for up to 20 days did not affect the early dedifferentiation usually observed in cultured chondrocytes. However, lipid droplets accumulated in DN-Erg chondrocytes, suggesting adipocyte emergence. Transcriptomic analysis using a DNA microarray, validated by quantitative RT-PCR, revealed strong differential gene expression, with a decrease in chondrogenesis-related markers and an increase in adipogenesis-related gene expression in cultured DN-Erg chondrocytes. These results indicate that Erg is involved in either maintaining the chondrogenic phenotype *in vitro* or in cell fate orientation. Along with the *in vitro* studies, we compared adipocyte presence in wild-type and transgenic mice skeletons. Histological investigations revealed an increase in the number of adipocytes in the bone marrow of adult DN-Erg mice even though no adipocytes were detected in embryonic cartilage or bone. These findings suggest that the Ets transcription factor family may contribute to the homeostatic balance in skeleton cell plasticity.

## Introduction

Chondrocytes, osteoblasts, fibroblasts, adipocytes and skeletal myoblasts are highly specific cell types derived from multipotent mesenchymal stem cells (MSC) through a specific differentiation pathway [1], [2], [3], [4], [5]. Although MSCs are fully committed to a developmental lineage, several studies have shown that MSC-derived cells can switch to another cell lineage or return to an uncommitted developmental stage [6], [7], [8], [9], [10]. Likewise, the signalling molecules and pathways leading to transdifferentiation (i.e. lineage reprogramming) remain poorly defined.

Chondrogenesis is a tightly regulated process that is initiated by the condensation of committed MSCs, followed by differentiation into chondrocytes and the expression of cartilage-specific markers [11], [12], [13]. Each specific differentiation program of MSC-derived cell types is harmoniously and dynamically controlled by several specific signal transduction (cytokines, growth factors and extracellular matrix molecules) and transcription factors [14], [15]. The osteochondrogenic state is regulated by two master transcription factors: Sox9 (Sex determining region Y-box9) and Runx2 (Runt-related transcription factor 2) essential for the determination and maturation of chondrocytes and osteoblasts, respectively [16], [17]. Several molecular players have been identified, including the transcription factors NF-κB, C/EBPβ, ETS, Runx2, and hypoxia-inducible factor-2α, all of which are involved in cartilage formation [18], [19], [20]. Among them, we and others have shown that the *Ets-related gene* (*Erg*) is expressed during the earliest events of skeletal formation and is associated with precartilaginous condensation and chondrogenic differentiation [21], [22], [23], [24]. The Erg transcription factor belongs to the ETS family of DNA-binding proteins [25]. Several members of the ETS family are involved in a variety of cellular and developmental processes. In skeletal formation, the *Erg* gene is the earliest ETS member family expressed in cartilage during embryonic development followed by *Fli1*, *Ets-2* and *Pea3* in a lesser extend [24], [26]. This family of transcriptional regulators shares a highly conserved 85 amino-acid DNA-binding domain (ETS domain) that specifically recognises DNA over an 11 bp sequence centred around a consensus core sequence, 5′-GGAA/T-3′ [27].

To explore the roles Erg may play in the chondrogenesis process or in chondrogenic maturation, we established a transgenic mouse model that overexpresses a dominant negative fragment of the Erg protein (DN-Erg), specifically restricted to the ETS domain. The transgene construct is specifically expressed in chondrocytes during cartilage formation in embryos because it is under the control of the *collagen II* (*Col2a1*) promoter and competes with endogenous wild-type Erg protein functions. However, since other ETS family genes, such as *Fli1*, *Ets2* and *Pea3* are also expressed, to a lesser extent, in cartilage, we cannot rule out that the binding of these transcription factors is also involved. Transgenic mouse embryos and newborns have no obvious malformations, but clinical early-ageing processes, including hyperlordosis/hyperkyphosis and reduced mobility, are observed during the first 6 months post natum (unpublished data). This manifestation of early ageing indicates that the Erg transcription factor is involved in the regulation of various genes affecting cartilage formation and skeletogenesis.

In this study, to explore the physiological roles of Erg proteins in the maintenance of the chondrocyte phenotype, we isolated chondrocytes from the rib cage of embryos of wild-type (wt) and DN-Erg transgenic mice and cultured them in monolayers. We observed that transgene expression was correlated with the accumulation of lipid droplets in cultured chondrocytes compared to wt. To determine the differentially expressed gene profile during the dedifferentiation process from monolayer-cultured wt and DN-Erg chondrocytes, we used DNA microarray analysis to study the transcriptome modifications in chondrocytes of wt and DN-Erg transgenic mice during culture. Among functional categories accounting for most genes with altered expression in cultured DN-Erg chondrocytes, the adipocyte pathway genes were upregulated.

In addition, because the phenotype of transgenic mice overexpressing DN-Erg was associated with early-ageing skeleton phenotypes and ageing is associated with decreased bone marrow cellularity and increased bone marrow fat, we performed a histological comparison of morphological features of bone marrow from adult mice femur and showed a dramatic increase in adipocytes in DN-Erg transgenic mice.

The data presented here demonstrate that monolayer-cultured DN-Erg chondrocytes spontaneously underwent adipocyte differentiation, suggesting that Erg is involved in the differentiation plasticity of chondrocytes.

## Materials and Methods

### Cell cultures

Chondrocytes of murine embryos were isolated from the ribs of 18.5 days post-coitum (E18.5) mice according to the protocol described in [28], [29]. Mouse care and treatment were conducted in accordance with institutional guidelines in compliance with national law and policies. This study was specifically approves by our local ethics committee (Authorisation no. CEEA 13/2009 issued by the Comite d'Ethique en Experimentation Animale, Nord-Pas-de-Calais). Chondrocytes were seeded in 6-well culture plates at 10^6^ cells/well and were grown for 20 days. Cells were cultured in DMEM Nutrient Mixture F-12 Ham (Sigma) supplemented with 10% foetal bovine serum, 1% gentamicin, and 1% glutamine. The culture medium was replaced every two days.

### Cytochemical analyses

#### Alcian blue staining

Cultures were washed with PBS before fixing with methanol for 10 min. After rinsing with PBS, cultures were stained overnight with Alcian blue solution (pH 2.5, 3% glacial acetic acid). Cells were washed three times with glacial acetic acid (4%) and twice with sterile distilled water.

#### Oil red O staining

Cells were washed twice with PBS and fixed with 10% formalin (pH 7.4) for at least 1 h at room temperature. After washing with 60% isopropanol, the cells were stained for 10 min at room temperature with filtered Oil red O/60% isopropanol solution. The cells were washed twice with distilled water. Red-stained adipocytes were observed under a light microscope.

### Histological analysis

Whole skeletons of E18.5 embryos were stained with Alizarin red S and Alcian blue [30]. Ribs and legs of 18.5 day embryos and of 40 week-old mice were dissected, fixed and embedded in paraffin. Paraffin blocks were prepared using standard histological procedures. The resulting serial sections (5–6 µm thickness) were stained with hematoxylin and eosin or Sudan black B, as indicated.

Adipocyte number was quantified by three different observers looking at five different fields per section at least three different mice. The result was expressed as the mean of total adipocyte number per square millimeter of marrow tissue area in the analysed fields.

### Immunohistochemistry

According to the provided protocol for immunohistochemistry, sections were demasked by treatment with xylene twice for 5 min at 37°C, rehydrated through graded ethanols and were incubated with rabbit anti-mouse adiponectin Acrp30 (N-20, sc-17044-R from Santa Cruz Biotechnology, dilution 1/100). Antigen retrieval was conducted in sodium citrate buffer pH 6 for 30 minutes at 95°C. A biotinylated goat anti-rabbit IgG antibody (Vector), diluted 1∶1000, was used as the secondary antibody with PBS for 1 hour at room temperature. Antigen immunolocalization was analysed using DAB peroxidise substrate (vector labs) according to manufacturer's instructions. Negative controls were realized by omitting primary antibodies. Counterstaining was done with hematoxylin.

### RNA preparation

Total RNA was isolated using the Nucleospin RNA II System (Macherey-Nagel GmbH &Co., Düren, Germany) according to the manufacturer's protocol. The RNA was eluted with 50 µl of RNase-free water. RNA integrity and purity were verified using the Agilent Bioanalyzer system (Agilent Technology).

### PCR and quantitative real-time PCR (RT-qPCR)

For PCR, 1 µg of total RNA was first reverse-transcribed using Superscript II reverse transcriptase (Invitrogen), random hexamers (Roche), and dNTPs at 42°C for 1 h. For non-quantitative PCR, the High Fidelity PCR Master kit (Roche) was used according to the manufacturer's protocol. Amplification conditions were adjusted to be within the linear range. Quantitative real-time PCR (RT-qPCR) was performed on a LightCycler (Roche Diagnostics) using the LightCycler FastStart DNA Master SYBR Green kit (Roche Diagnostics) according to the manufacturer's instructions. The PCR primers were designed to amplify cDNA fragments ranging in size from 150 to 400 bp and are listed in Table 1. Gene expression levels in each sample were determined using the comparative Ct method (after validation assays for each gene primer set), using the hypoxanthine-guanine phosphoribosyltransferase (*HPRT*) gene as an endogenous control. The wt control was set to 1 and expression data are presented as bar graphs of the mean values with their SD.

**Table 1:** Primer sequences of genes investigated in this study.

Symbol	Full name	Accession #	Forward (F) and Reverse (R) primer sequence
Adamts5	Disintegrin-like and metallopeptidase (reprolysin type) with thrombospondin type 1 motif, 5	NM_011782	F: 5′-ATGCAGCCATCCTGTTCAC-3′
			R: 5′-CATTCCCAGGGTGTCACAT-3′
Adipoq	Adiponectin, C1Q and collagen domain containing	NM_009605	F: 5′-CAGGCATCCCAGGACATCC-3′
			R: 5′-CCAAGAAGACCTGCATCTCCTTT-3′
Col2a1	Collagen, type II, alpha 1	NM_001113515	F: 5′-GGTGGCTTCCACTTCAGCTAT-3′
			R: 5′-TCATTGGAGCCCTGGATGAG-3′
Col10a1	Collagen, type X, alpha 1	NM_009925	F: 5′-TTCTCCTACCACGTGCATGTG-3′
			R: 5′-AGGCCGTTTGATTCTGCATT-3′
CtsC	Cathepsin C	NM_009982	F: 5′-CCAACTGCACCTACCCTG-3′
			R: 5′-CTGAACGGTATTGATGGCCT-3′
CtsS	Cathepsin S	NM_021281	F: 5′-TGGTGACGAAGATGCCCTGAAAGA-3′
			R: 5′-TGCCATCAAGAGTCCCATAGCCAA-3′
Erg	Avian erythroblastosis virus E-26 (v-ets) oncogene related	NM_133659	F: 5′-GTGGGCGGTGAAAGAATATGG-3′
			R: 5′-CTTTGGACTGAGGGGTGAGG-3′
DN-Erg	Transgen Dominant-Negatif Erg	-	F: 5′-ACCCACAGAAGATGAACTTTG-3′
			R: 5′-GGATCCACTAGTTCTAGAGG-3′
Fabp4	Fatty acid binding protein 4, adipocyte	NM_024406	F: 5′-CAAAATGTGTGATGCCTTTGTG-3′
			R: 5′-GGCTCATGCCCTTTCATAAAC-3′
Hprt	Hypoxanthine guanine phosphoribosyl transferase	NM_013556	F: 5′-GCTGGTGAAAAGGACCTCT-3′
			R: 5′-AAGTAGATGGCCACAGGACT-3′
Matr3	Matrin3	NM_010770	F: 5′-TTACCAGCACCCAGATTTCC-3′
			R: 5′-TGGAGCAAGTCACAGTCGTC-3′
Mmp3	Matrix metallopeptidase 3	NM_010809	F: 5′-TGACCCACATATTGAAGAGC-3′
			R: 5′-ACTTGACGTTGACTGGTGTC-3′
Mmp9	Matrix metallopeptidase 9	NM_013599	F: 5′-ACTCACACGACATCTTCCAG-3′
			R: 5′-AGAAGGAGCCCTAGTTCAAG-3′
Mmp13	Matrix metallopeptidase 13	NM_008607	F: 5′-TTTATTGTTGCTGCCCATGA-3′
			R: 5′-TTTTGGGATGCTTAGGGTTG-3′
Plin	Perilipin 1	NM_001113471	F: 5′-TGCTGGATGGAGACCTC-3′
			R: 5′-ACCGGCTCCATGCTCCA-3′
Pparg	Peroxisome proliferator activated receptor gamma	NM_011146	F: 5′-GCATCAGGCTTCCACTATGGA-3′
			R: 5′-AAGGCACTTCTGAAACCGACA-3′
Runx2	Runt related transcription factor 2	NM_001145920	F: 5′-GAGGCCGCCGCACGACAACCG-3′
			R: 5′-CTCCGGCCCACAAATCTCAGA-3′
Scin	Scinderin	NM_009132	F: 5′-AACAGTGGTAGAGTCCAGATT-3′
			R: 5′-GTGATAGATGCCAGGTTCCTC-3′
Sox9	SRY-box containing gene 9	NM_011448	F: 5′-TGGCAGACCAGTACCCGCATCT-3′
			R: 5′-TCTTTCTTGTGCTGCACGCGC-3′

### Microarray Analysis

Hybridisation was carried out following the Two-Colour Microarray-Based Expression Analysis protocol (Agilent Technologies). For each sample, 1 µg of total RNA per sample was divided into two equal aliquots to enable technical replication known, as a dye-swap hybridisation, and amplified. Dye-swap hybridisations were performed by reversing the dyes for each of the RNA samples. The reverse transcription and the labelling were conducted using the protocol recommended by Agilent (Agilent Low RNA Input Fluorescent Linear Amplification kit). Whole Mouse Genome Oligo Microarrays from Agilent Technologies were used and hybridisations were performed for 17 h at 65°C (Gene Expression Hybridization kit, Agilent Technologies). Slides were washed and scanned using Innoscan 700 (Innopsys) and raw data were processed using the Limma package (Linear Models for Microarray Data) running under the statistical language R. A normalisation protocol consisting of a within-array loess normalisation to correct for dye and spatial effects was applied on the median or mean intensities of the spots. After normalisation, identification of statistically significant deregulation was performed using a moderated Student's *t*-test with empirical Bayes shrinkage of standard errors. We selected genes whose expression was significantly upregulated (mean log-ratio >3.3x; adjusted p-value <0.005) or downregulated (mean log-ratio <−3.3x; significant at the adjusted p-value <0.005 level). The data were further analysed using MultiExperiment Viewer (version 4.5), Fatigo and Pathway-Express. The latter was used to predict molecular signalling pathways by assessing an impact factor that accounts for contribution to the proportion of differentially regulated genes in a given pathway.

## Results

### Morphological phenotype of adipocytes in monolayer-cultured DN-Erg chondrocytes

**Figure 1:**
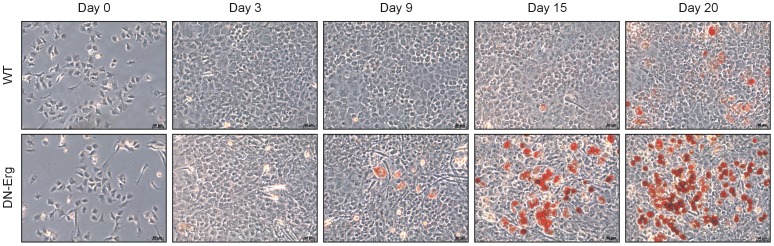
Morphological changes in wt and DN-Erg E18.5 chondrocytes in culture. Chondrocytes from freshly isolated from ribs of wt and DN-Erg transgenic mouse embryos (at E18.5) were cultured for up 20 days and stained with Oil red O. Phase-contrast images at days 0, 3, 9, 15 and 20 (with day 0, the day of plating) are shown (Scale bar, 50 µm).

To investigate how modified Erg function (via dominant negative transgene expression) affects chondrocyte phenotype, we isolated chondrocytes from the ribs of wt or DN-Erg E18.5 embryos and cultured them in a conventional monolayer culture. After plating chondrocytes in culture dishes (designated as day 0), cells showed a polygonal shape until confluence (Fig. 1, day 0 to day 3). Thereafter, the morphology of DN-Erg chondrocytes changed rapidly and dramatically. Some DN-Erg cells became enlarged fibroblast-like cells and accumulated vesicles. These vesicles were strongly stained with Oil red O, suggesting they were lipid droplets. Compared with DN-Erg cells, which accumulated lipid droplets from day 9 to day 20 (Fig. 1 and Fig. S1), wt chondrocyte cultures only just begun to show weak Oil red O staining as of day 20. Using an Alcian blue stain as a chondrocyte-specific matrix stain, DN-Erg cultures showed a decrease in cartilage matrix deposition in comparison to wt cultures (Fig. S2). These morphological and cytochemical observations showed that monolayer-cultured DN-Erg chondrocytes display an adipocyte-like phenotype, suggesting that DN-Erg transgene expression favours an adipocyte-like phenotype in monolayer culture over time.

### Decrease in chondrocyte marker expression and detection of adipocyte-related genes in cultured transgenic DN-Erg chondrocytes

**Figure 2:**
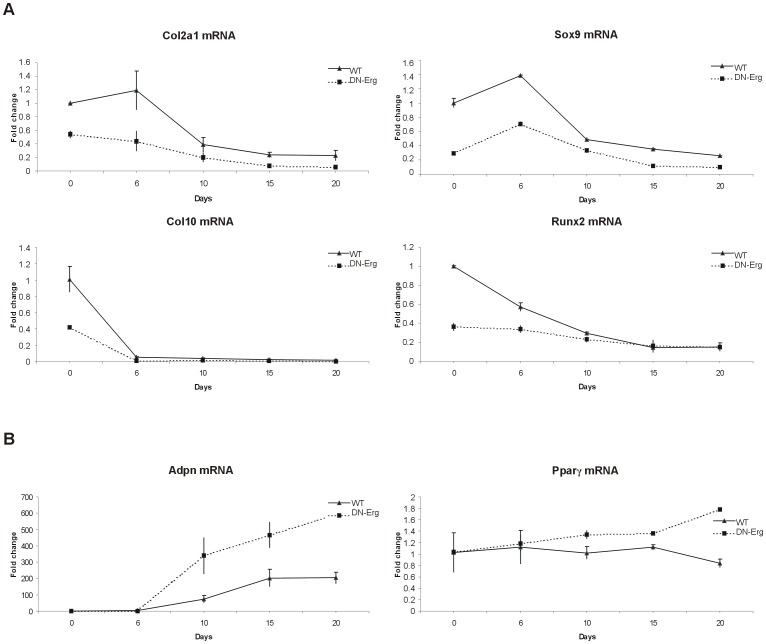
Expression of chondrogenic and adipogenic markers during monolayer culture of wt and DN-Erg chondrocytes. A. Expression of chondrogenic genes *Col2a1*, *Sox9*, *Col10*, and *Runx2*. Gene expression was evaluated by RT-qPCR. B. Expression of adipogenic genes *Adpn*, and *Pparγ*. Gene expression was evaluated by RT-qPCR. The reported target gene: *Hprt* transcript ratio in chondrocytes was normalised to the target gene:*Hprt* transcript ratio (set to 1) of freshly plated wt chondrocytes (day 0). Data represent the mean of at least 2 independent chondrocyte cultures from 2 distinct mice for each genotype.

We observed great morphological differences between DN-Erg and wt chondrocytes over the 20 day culture period. To characterize the adipocyte-like phenotype of DN-Erg chondrocytes, we paid special attention to the expression of specific chondrogenic and adipogenic markers using quantitative real-time PCR (Fig. 2). Total RNA was collected from wt and DN-Erg chondrocytes after 0, 6, 10, 15 and 20 days of culture.

We studied the expression of four major chondrocyte markers, namely *Col2a1*, *Sox9*, *Col10* and *Runx2* (Fig. 2A). As expected, in wt chondrocytes, *Col2a1* and *Sox9* showed a peak in expression during the first 6 days of culture, followed by a dramatic decrease. The expression level of these two markers was 2-fold and 4-fold lower, respectively, in DN-Erg chondrocytes compared with wt chondrocytes. Likewise, *Col10* and *Runx2* expression, which were 2- to 3-fold downregulated in DN-Erg, decreased rapidly in both chondrocyte cultures within 6 and 10 days of culture, respectively, and remained at low levels throughout the rest of the culture period. We also verified DN-Erg expression. As expected, since the transgene is under *Col2a1* promoter control, the transgene expression was expressed during the culture, and its pattern was similar to the *Col2a1* pattern (Fig. S3).

Because Oil red O staining revealed lipid vesicles in cultured DN-Erg cells, we assayed the expression of adipocyte markers, such as the *adiponectin* (*Adpn*) and *peroxisome proliferator-activated receptor gamma* (*Pparγ*) genes (Fig. 2B). In both chondrocyte cultures, we detected *Adpn* transcripts at very low levels until day 6 of culture. *Adpn* expression was upregulated at day 20 compared to day 0, when mRNA levels were 200- and 600-fold greater in wt and DN-Erg chondrocytes, respectively. Although *Pparγ* mRNA level remained constant throughout wt chondrocyte culture, it increased in DN-Erg chondrocytes after 6 days of culture.

Taken together, these results validated the observed phenotypic changes. DN-Erg chondrocytes cultured as monolayers experienced a dramatic decrease in the expression of chondrocyte-specific markers and a strong emergence of adipocyte-specific markers.

### Comparative differential transcriptomic analysis of monolayer-cultured wt and DN-Erg chondrocytes

To investigate and compare all the modulated genes in monolayer-cultured DN-Erg and wt chondrocytes over time, we monitored the change in genome-wide expression patterns with a global differential approach using whole Mouse Genome Oligo Microarrays (Agilent). Using the monolayer cultures, total RNA isolated from day 0 to day 20 were hybridised to a microarray containing 44,000 probe sets representing nearly 41,000 mouse genes. Comparative microarray analyses were done using Mapix 3.2 (Innopsys). Because the number of differentially expressed genes was high, we chose a cut-off value of a 10-fold change in expression and an adjusted p-value of <0.005.

**Figure 3:**
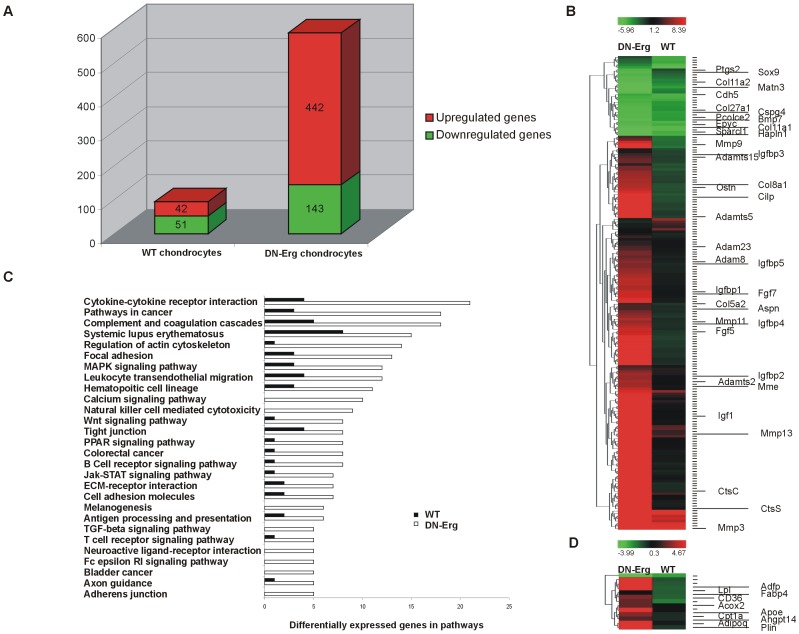
Microarray analysis and gene ontology analysis of signalling pathways. A. Numbers of genes with differential expression between monolayer culture day 0 and day 20 in wt and DN-Erg embryo (E18.5) chondrocytes. Probe sets were filtered according to a 10-fold change cut-off. B. Hierarchical Clustering (HCl) diagram with clusters genes corresponding to the “extracellular matrix”, “metallopeptidase activity”, “Cartilage condensation and development”, “Ossification” annotations. C. Major signalling pathways predicted using Pathway-Express. Pathways listed are pathways with at least 5 or more genes which expression was modified during culture, as determined by Pathway Express. D. Hierarchical Clustering (HCl) tree with clusters of “Lipid metabolism process” and “Lipid transport” genes.

The results show that 93 transcribed genes (42 upregulated and 51 downregulated) were significantly modified 10-fold or more between day 0 and day 20 in cultured wt chondrocytes (Fig. 3A and Table S1), 585 genes (442 upregulated and 143 downregulated) were modified between day 0 and day 20 in cultured DN-Erg chondrocytes (Fig. 3A and Table S2). Finally, even with the stringent cut-off criterion (i.e. value of 10-fold or more), the number of modified genes was six times greater in DN-Erg chondrocytes than in wt chondrocytes between day 0 and day 20 of culture.

To classify the genes whose expression had been modified, further examination was performed on the basis of Gene Ontology (GO) annotations (Fig. 3B). We first focused on genes classified by the following functional annotations from the Gene Ontology Database: “extracellular matrix region”, “metallopeptidase activity”, “cartilage condensation and development”, and “ossification” annotations. We then used hierarchical clustering to group together genes with similar expression patterns (Table S3). This process resulted in a selection of 190 upregulated or downregulated genes coding for collagens, proteoglycans, matrix-modifying enzymes, growth factors and transcription factors (Fig. 3B). Among these 190 genes, 9 genes whose expression is known to decrease during chondrocyte dedifferentiation were assayed and found to be dramatically downregulated in cultured DN-Erg chondrocytes: *scinderin* (*Scin*), *hyaluronan and proteoglycan link protein1* (*Hapln1*), *matrilin 3* (*Matn3*), *SPAR like 1*, *Col11a2*, *epiphycan* (*Epyc*), *col27a1*, *fibroblast growth factor receptor* (*Fgfr3*) and the transcription factor Sox9, which showed decreased expression in the previous experiment (Fig. 2B). Likewise, the following genes were substantially upregulated: matrix metallopeptidase and aggrecanase (*Adamts5*, *Adamts2*, *Adamts15*, *Mmp 13*, *Mmp3*, *Mmp9*, and *Mmp11*).

To identify functionally related patterns from the list of genes differentially expressed between day 0 and day 20, we carried out a pathway analysis and compared the relative distribution of functions (Fig. 3C). Genes modified between day 0 and day 20 in DN-Erg chondrocytes were involved in several functional pathways. In accordance with what we observed in the cultured DN-Erg cell plate, we noticed clusters of genes involved in the ‘PPAR signalling pathway’ and in ‘adipocyte differentiation’ (from the Gene Ontology Database) (Table S4). Nine transcripts (*Adpn*, periplin (*Plin*), fatty acid binding protein 4 (*Fabp4*), lipoprotein lipase (*Lpl*), carnitine palmitoyltransferase 1a liver (*Cpt-1a*), acyl-Coenzyme A oxidase 2 branched chain (*Acox2*), angiopoietin-like 4 (*Angptl4*), CD36 antigen (*CD36*), adipose differentiation related protein (*Adfp*)) were associated with the ‘PPAR signalling pathway’ (Fig. S4) and were significantly upregulated in DN-Erg chondrocytes cultured for 20 days (Fig. 3D and Table S4).

### Validation of changes in gene expression by real-time PCR

To confirm the observed differences in gene expression between day 0 and day 20 of cultured DN-Erg and wt cells by microarray assay, we performed a quantitative RT-PCR (RT-qPCR) analysis on 11 additional selected genes products (Table 2) in addition to *Sox9* and *Adpn* genes (Fig. 2). In all cases, the direction of change in expression was concordant between the microarray and RT-qPCR results, although absolute values of the microarray-estimated fold change and RT-qPCR-calculated fold change were different (e.g. *Matn3*). Nevertheless, RT-qPCR results were in line with microarray data and confirmed the loss of the chondrocyte phenotype and the dramatic upregulation of adipocyte markers in cultured DN-Erg chondrocytes.

**Table 2:** Correlation of microarray data and RT-qPCR analysis.

		Microarray		Real-Time qPCR	
		(Fold-change between day 20 vs day 0 of culture)		(Fold-change between day 20 vs day 0 of culture)	
Sequence Name	Accession	WT	DN-ERG	WT	DN-ERG
***Cartilage Development***					
Scinderin	NM_009132	−2	−71	−14	−795
Matn3	NM_010770	−7	−39	1	−2
***Metalloproteinases***					
ADAMTS5	NM_011782	2	49	4	56
MMP3	NM_010809	40	284	13	87
MMP9	NM_013599	−1	26	1	137
MMP13	NM_008607	11	49	10	71
***Cathepsine***					
CTS C	NM_009982	4	131	39	317
CTS S	NM_021281	6	288	52	1116
***Adipocyte Differentiation***					
Plin	NM_175640	4	64	3	16
FABP4	NM_024406	3	16	146	284
Adipoq	NM_009605	9	84	200	467

Comparison of fold change in the expression of 11 genes implicated in chondrogenesis or adipogenesis as determined by microarray analysis and RT-qPCR.

Fold change between day 0 and day 20 observed by microarray-analysis and RT-qPCR for wt and DN-Erg chondrocytes are shown. Expression levels of target genes obtained by RT-qPCR were normalised to *Hprt*. Upregulation is indicated by positive values and downregulation is indicated by negative values.

### Predominant adipogenesis in bone marrow of adult DN-Erg mice

**Figure 4:**
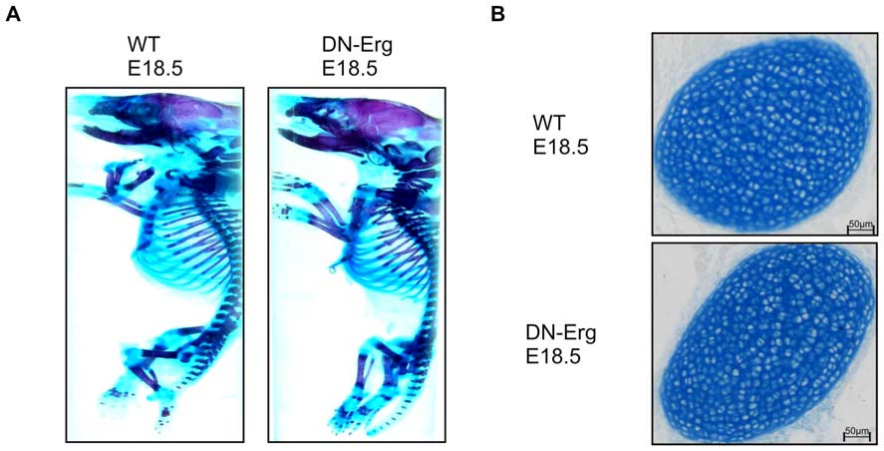
Histological examination of 18.5 day-old embryos. A. Skeletal and cartilage preparations of wt (left) and DN-Erg (right) mice at E18.5. Cartilage stained with Alcian blue, bone with Alizarin red. DN-Erg E18.5 embryos did not show any overt abnormalities in cartilaginous or skeletal development. B. Distribution of chondrocytes on sections of rib. Paraffin-embedded sagittal sections of wt (left) and DN-Erg (right) newborn mice were stained with Alcian blue. Bars = 50 µm.

Since the cultured primary chondrocytes were isolated from the ribs of 18.5 day-old wt and DN-Erg embryos, we compared the cartilaginous framework phenotype of wt and DN-Erg embryos (Fig. 4A and unpublished data). The ribs were morphologically homogenous in appearance and consisted only of chondrocytes evenly distributed in extracellular matrix (Fig. 4B).

**Figure 5:**
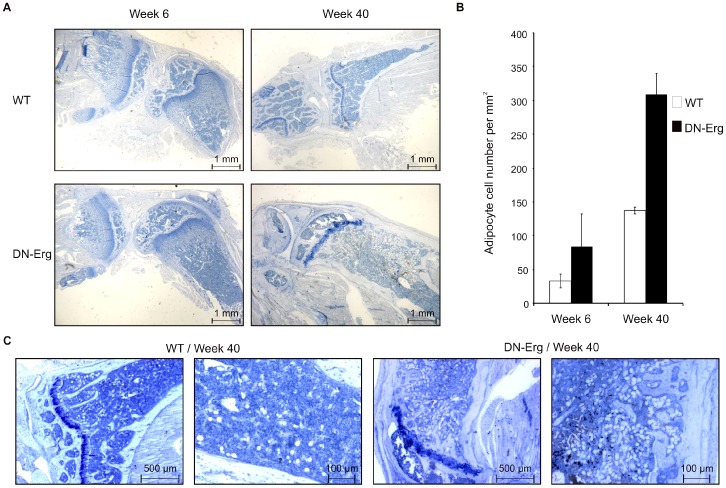
Cytological analysis and adipocyte quantification of femoral bone marrow of wt and DN-Erg mice. A. Sections of femorotibial joint of week 6- and week 40- wt or DN-Erg mouse. Bars = 1 mm. B. Quantification of adipocytes in bone marrow of DN-Erg and wt femoro-tibial section. The result was expressed as the mean of total adipocyte number per square millimeter of marrow tissue area in the analysed fields of at least three different mouse. C. Cytological examination of bone marrow in a femur section of 40 week-old wt and DN-Erg mice. Left, Magnification: ×2.5. Right: higher magnification view (×10).

The particular phenotype of DN-Erg transgenic mice, such as early-ageing processes, including hyperlordosis/hyperkyphosis and reduced mobility, appeared with age and was associated with an arthritis-like phenotype (unpublished data). Given these results, we were interested in features of bone marrow from tibia and femur of transgenic mice. Histological examination of limb sections stained with hematoxylin/eosin showed the distinct appearance of adipocytes occupying the marrow cavity (Fig. 5A and Fig. S5A). A clear increase in the number of adipocytes present in the bone marrow of mice was observed upon ageing (Fig. 5A, S5A). Adipocyte densities were significantly higher in DN-Erg compared to wt mice at weeks 6 and weeks 40 (Fig. 5B, 5C). Although adipocytes were readily distinguishable from other cell types present in bone marrow by their morphology (Fig. 5C), Sudan black B staining and immunocytochemistry staining of adiponectin were performed to confirm our observations (Fig. S5B and S5C).

Taken together, these histological investigations of bone marrow from adult mice femur revealed that ETS transcription factor family may contribute to the homeostatic balance in skeleton cell plasticity *in vivo*.

## Discussion

*Erg* expression is associated with early events of cartilage formation during development [21], [24], [31], but its physiological role in this mechanism has been poorly understood. Several specific transcription factors (such as Nkx3.2, Sox9, etc.) have been shown to be involved in the maintenance of the chondrogenic state [32], [33] or in the promotion of transdifferentiation into an adipogenic state [34]. In the present study, we showed that Erg is an important component in the maintenance of the chondrogenic phenotype *in vitro*, and in the healthy ageing of the skeleton. However, since other ETS family genes are also expressed, to a lesser extent, in cartilage, we cannot rule out that the binding of these transcription factors is also potentially disturbed.

Chondrocytes that are grown in a standard monolayer culture are known to lose their phenotype after a few passages [35]. Furthermore, studies have shown that chondrocytes of the mouse process may transdifferentiate into adipocytes under culture conditions and accumulate lipid droplets [8], [34]. In our study, we observed that chondrocytes isolated from embryos of a transgenic mouse model (expressing a dominant negative form of Erg during cartilage formation and cultured *in vitro*) and a wt mouse rapidly lose their chondrogenic phenotype. In contrast, only DN-Erg chondrocytes expressed the adipogenic phenotype over time. Significant morphological differences, such as cell morphology and lipid accumulation, were observed in DN-Erg chondrocytes stained in monolayer culture. These observations were confirmed by a comparative transcriptomic analysis of cultured DN-Erg chondrocytes. A high cut-off value was needed to analyse cDNA microarray because of the high number of deregulated genes. GO analysis identified specific groups of regulated gene products associated with a loss of the chondrogenic phenotype and the appearance of adipogenic differentiation markers. Expression of several genes involved in chondrocyte differentiation and hypertrophic maturation dramatically decreased between day 0 and day 20 in DN-Erg compared with wt chondrocytes, whereas the expression of genes involved in matrix metalloproteinases (MMPs) and aggrecanases, leading to articular cartilage destruction, were upregulated. Moreover, the expression of several key features of osteoarthritis pathology [36] and pro-inflammatory mediators (e.g. cysteine proteases cathepsin S and cathepsin C [37]), were detected among the highly regulated genes during DN-Erg chondrocyte culture.

The expression of three master genes *Sox9*, *Runx2* and *Pparγ*, which are critical for phenotype determination at early stages of mesenchymal cells in cartilage formation, osteogenesis and adipogenesis, respectively, were affected in freshly isolated and in cultured DN-Erg chondrocytes. Knowledge on each transcription factor in the commitment and the maintenance of chondrogenic cell lineages suggests that their deregulation may contribute to the dedifferentiation of chondrocytes and the switch of chondrocytes into adipocytes. Sox9 plays an essential role in the promotion of chondrogenesis [38] and may maintain chondroblasts in an immature state. In addition, the transcription factor Runx2 is a critical enhancer of chondrocyte maturation and osteoblast differentiation. The expression of these two transcription factors was decreased by over 60% in DN-Erg chondrocytes relative to wt chondrocytes. Such low expression levels of key chondrogenesis regulators may explain the early dedifferentiation of cultured DN-Erg chondrocytes. Moreover, as for the depletion of Runx2 in chondrocytes [39], the competition of Erg with a transdominant negative protein favours the loss of the well-established chondrocyte phenotype and the emergence of adipocytes. To show the potent inhibition of the Erg transcription factor in the adipocyte differentiation process, we followed *Erg* expression in 3T3L1 pre-adipocytes cells (data not shown). The *Erg* gene is expressed at a very basal level in 3T3L1 cells, and its level did not change when adipocyte differentiation was induced. Moreover *Erg* overexpression did not affect adipogenenic differentiation of 3T3L1 cells (data not shown). Altogether, these results suggest that although the Erg protein may be involved in the inhibition of the transdifferentiation of chondrocytes into adipocytes, it was not associated with the adipogenic process.

The nuclear receptor Pparγ, mainly involved in the regulation of adipogenesis and in the expression of adipocyte-related differentiation marker genes, also plays an important, albeit complex, role in bone metabolism. On the one hand, Pparγ favours the differentiation of mesenchymal stem cells into adipocytes rather than osteoblasts or chondrocytes [40]; *Pparγ* overexpression has been reported to promote adipogenic differentiation in growth plate chondrocytes [34], but it has no effect on fully differentiated osteoblasts or osteoclasts [41]. On the other hand, *in vivo* Pparγ has been shown to be expressed and activated in articular chondrocytes. It is required for endochondral ossification and cartilage development, and has chondroprotective properties against osteoarthritis [42], [43], [44], [45]. Several studies have shown that Ppar*γ* is expressed in hypertrophic chondrocytes. Even if its role remains incompletely resolved, the expression of this transcriptional factor is involved in lipid and energy metabolism [46]. In the present study, *Pparγ* was expressed in wild-type and DN-Erg chondrocytes, and no difference in the level of *Pparγ* mRNA expression was found in freshly isolated chondrocytes. However, the expression of *Pparγ* and its target genes were progressively increased in DN-Erg chondrocytes during culture, which may affect chondrocyte features and accelerate the differentiation pathway to adipocytes.

The present results obtained with primary chondrocytes prompted us to compare skeletal features of transgenic mice with wt mice. It is well known that, with age, bone marrow becomes enriched into adipocytes [47], [48]. Moreover, age-related diseases such as osteoporosis and osteopaenia are described as accompanied by a pronounced accumulation of adipocytes in bone marrow [49], [50], [51], [52]. In contrast with adult wt mice, DN-Erg mice showed predominantly fatty marrow. The function and origin of fatty marrow are still largely unknown [53]. However, these little-studied cells play a more active role than just passively filling the bone cavity [54]. Given the abundant variety of adipokines secreted by adipocytes, the increase in adipocytes in the bone marrow helps to inhibit functions of other bone cells, including osteoblasts and hematopoietic stem cells [55], [56], and structural changes in the surrounding cartilage matrix, thereby disturbing bone regeneration [49], [57], [58], [59]. All these results raise the question of the origin of adipocytes in bone marrow. The bone marrow formation occurs post-natally via the invasion of capillaries through the cortical bone shaft. Downregulation of Sox9 in the hypertrophic zone of the growth plate is required for cartilage-bone transition and bone marrow formation [60]. In this context, our results suggest that DN-Erg transgene expression during chondrogenesis may affect skeletal plasticity and bone quality in adults.

In summary, the present study strongly suggests that the Erg transcription factor is involved in chondrogenesis and plays an important role in the maintenance of the chondrocyte pathway. The potential role of the Ets protein in skeletal cell plasticity was further highlighted by *in vivo* observations of accelerated formation of fatty marrow in adult DN-Erg mice. Finally, factors affecting cartilage formation may have a high incidence on the occurrence of ageing-related diseases.

## Supporting Information

Figure S1 **Chondrogenic phenotype was assessed by Alcian blue staining.** Chondrocytes were cultured for 15 days, then stained with Alcian blue and observed under a phase-contrast microscope at ×10 magnification.
(TIF)

Figure S2 **Oil red O staining of chondrocytes isolated from WT and DN-Erg E18.5 mice and cultured for 20 days.** Low-magnification; inset: ×10 magnification.
(TIF)

Figure S3 **PCR Analysis of mRNA levels in DN-Erg mice after 0, 6, 10, 15 and 20 days of culture.** The reported target gene (i.e. DN-Erg):*Hprt* transcript ratio at each time was determined par qPCR and was normalised to DN-Erg∶*Hprt* transcript ratio (set to 1) on day 0. ND = not determined.
(TIF)

Figure S4 **PPAR signalling pathway based on Pathway-Express.** In the network diagrams, the red boxes indicate genes whose mRNA levels changed during the 20 days of culture.
(TIF)

Figure S5 **Histological analysis of bone marrow and Adiponectin detection in femur sections of DN-Erg.** A. Cytological examination of bone marrow in femur sections of 6, 24 and 40 week-old wt and DN-Erg mice. Scale bar: 100 µm. B. Lipid staining with Sudan black B (Merck) was used to evaluate lipid droplets within *adipocytes* according to Soldani et al. [61] on section from femur of 40 week-old DN-Erg mice. Scale bar: 50 µm. C. Adiponectin immunocytochemical staining in bone marrow of 40 week-old DN-Erg mice femur. Scale bar: 50 µm.
(TIF)

Table S1 **List of genes with a 10-fold or more change in expression between day 0 and day 20 of culture in wt chondrocytes in microarray-derived data.**
(XLS)

Table S2 **List of genes with a 10-fold or more change in expression between day 0 and day 20 of cultured in DN-Erg chondrocytes in microarray-derived data.**
(XLS)

Table S3 **List of genes classified by the “extracellular matrix region”, “metallopeptidase activity”, “cartilage condensation and development”, “ossification” annotations in the Gene Ontology Database.**
(XLS)

Table S4 **List of genes classified by the “PPAR signalling” and “adipocyte differentiation” annotations in the Gene Ontology Database.**
(XLS)
