# Supplementary figures and images for: Increased Adipogenesis in Cultured Embryonic Chondrocytes and in Adult Bone Marrow of Dominant Negative Erg Transgenic Mice
Source: PLoS One. 2012 Nov 14;7(11):e48656. doi: 10.1371/journal.pone.0048656 (PMC3498236; doi:10.1371/journal.pone.0048656)

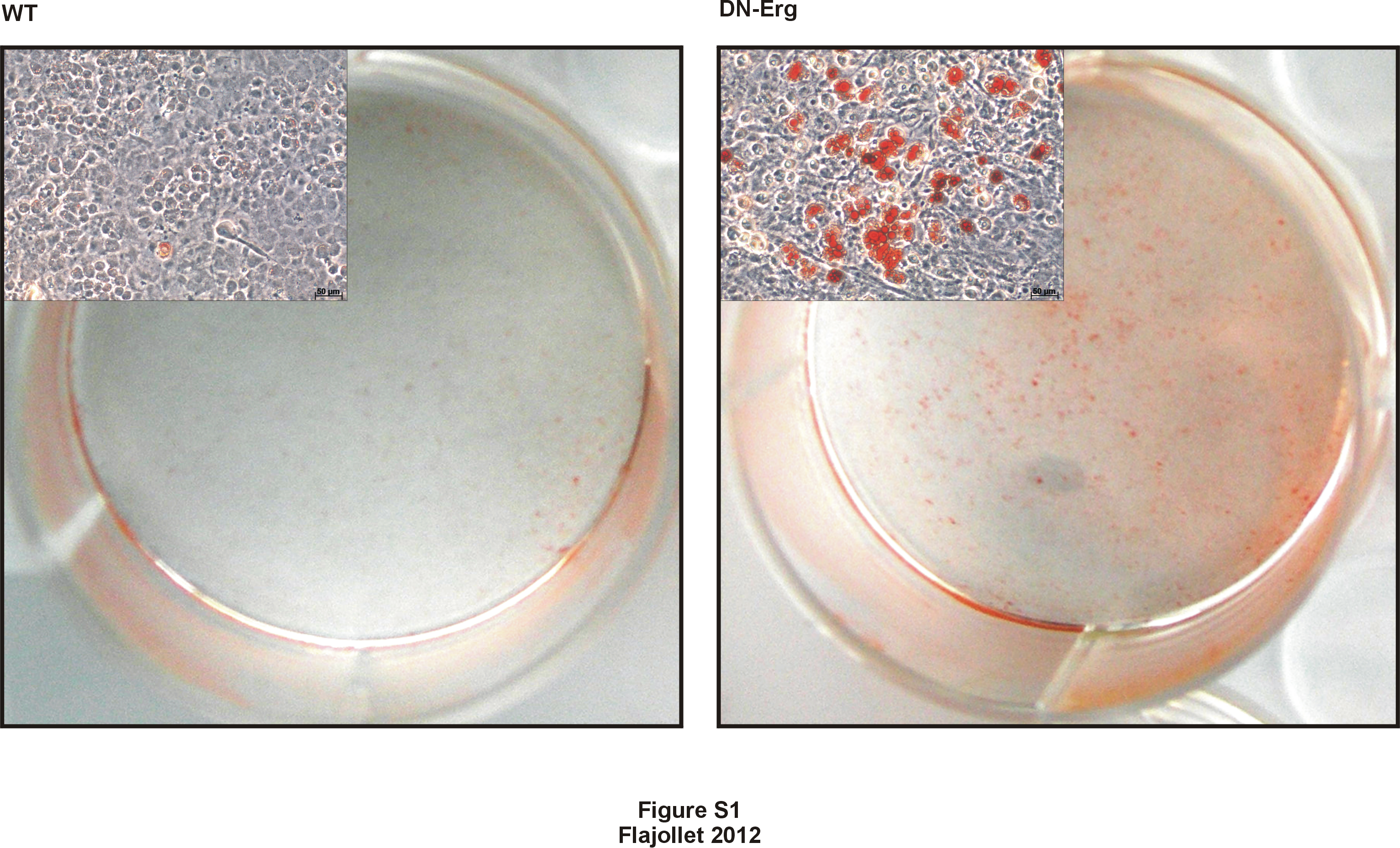

Supplement: Figure S1 — Chondrogenic phenotype was assessed by Alcian blue staining. Chondrocytes were cultured for 15 days, then stained with Alcian blue and observed under a phase-contrast microscope at ×10 magnification. (TIF) [file pone.0048656.s001.tif]

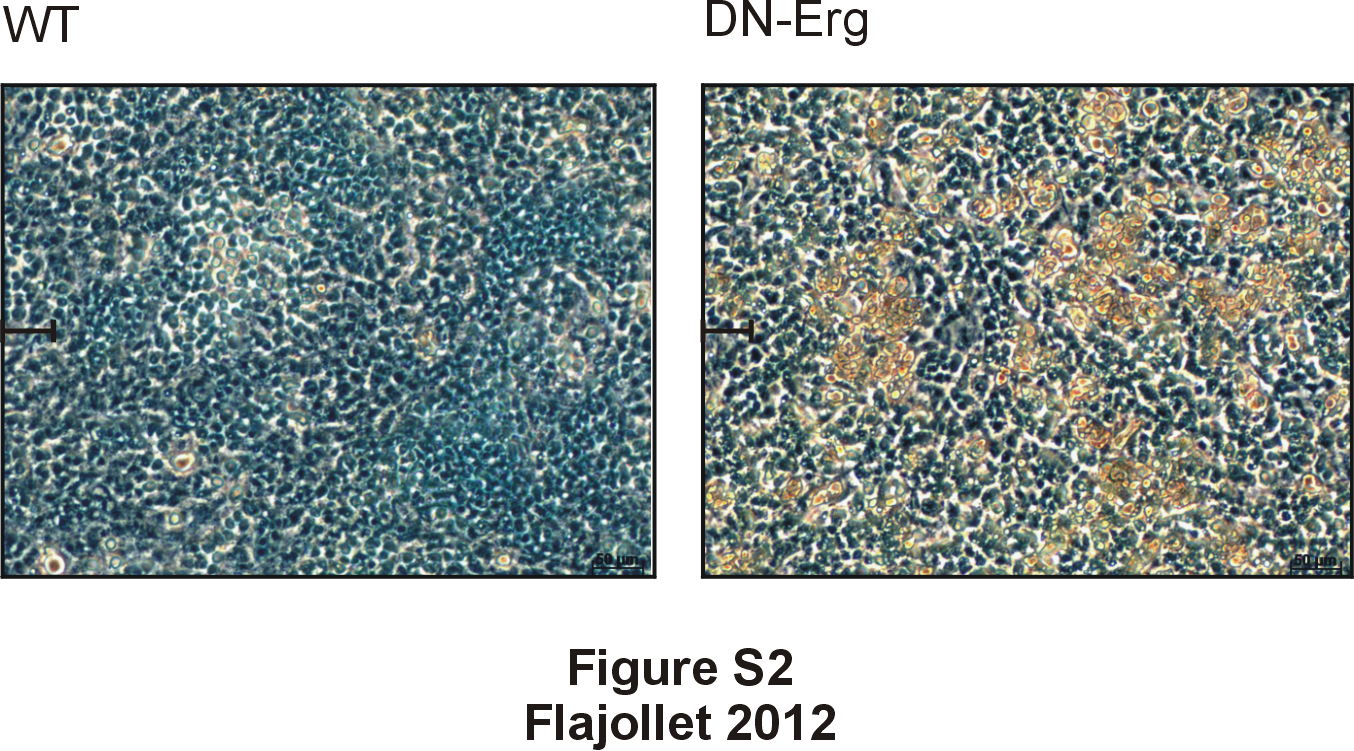

Supplement: Figure S2 — Oil red O staining of chondrocytes isolated from WT and DN-Erg E18.5 mice and cultured for 20 days. Low-magnification; inset: ×10 magnification. (TIF) [file pone.0048656.s002.tif]

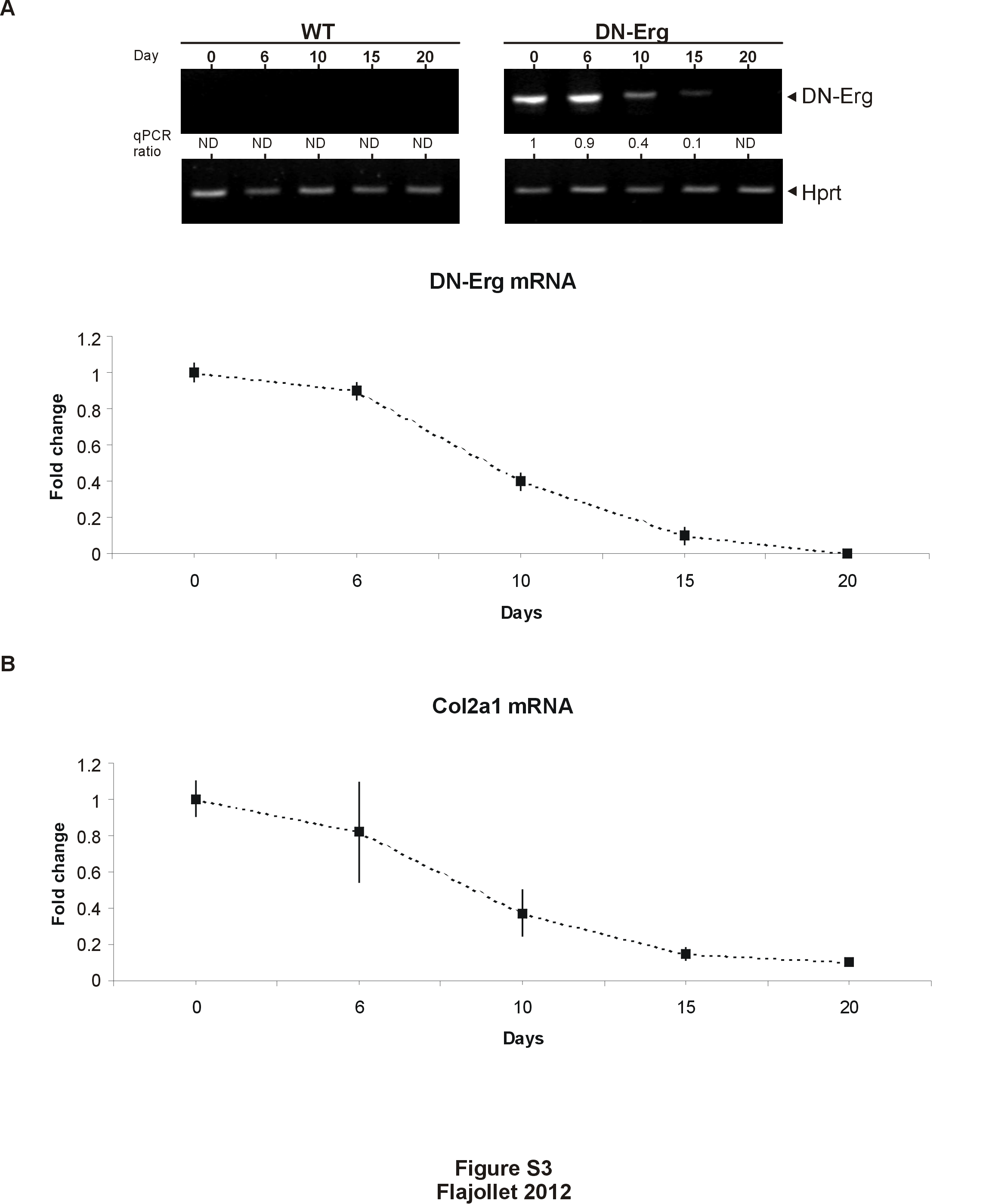

Supplement: Figure S3 — PCR Analysis of mRNA levels in DN-Erg mice after 0, 6, 10, 15 and 20 days of culture. The reported target gene (i.e. DN-Erg):Hprt transcript ratio at each time was determined par qPCR and was normalised to DN-Erg∶Hprt transcript ratio (set to 1) on day 0. ND = not determined. (TIF) [file pone.0048656.s003.tif]

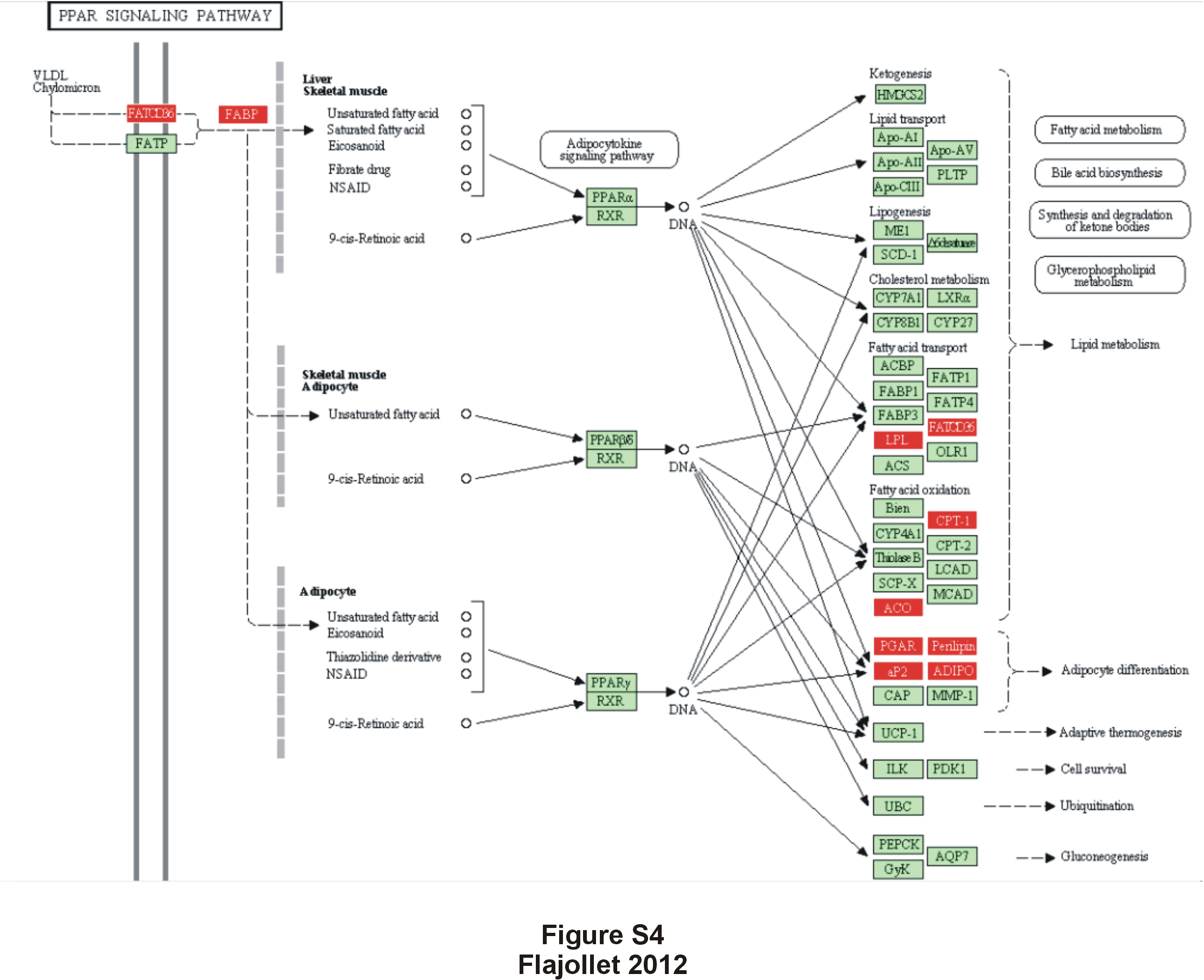

Supplement: Figure S4 — PPAR signalling pathway based on Pathway-Express. In the network diagrams, the red boxes indicate genes whose mRNA levels changed during the 20 days of culture. (TIF) [file pone.0048656.s004.tif]

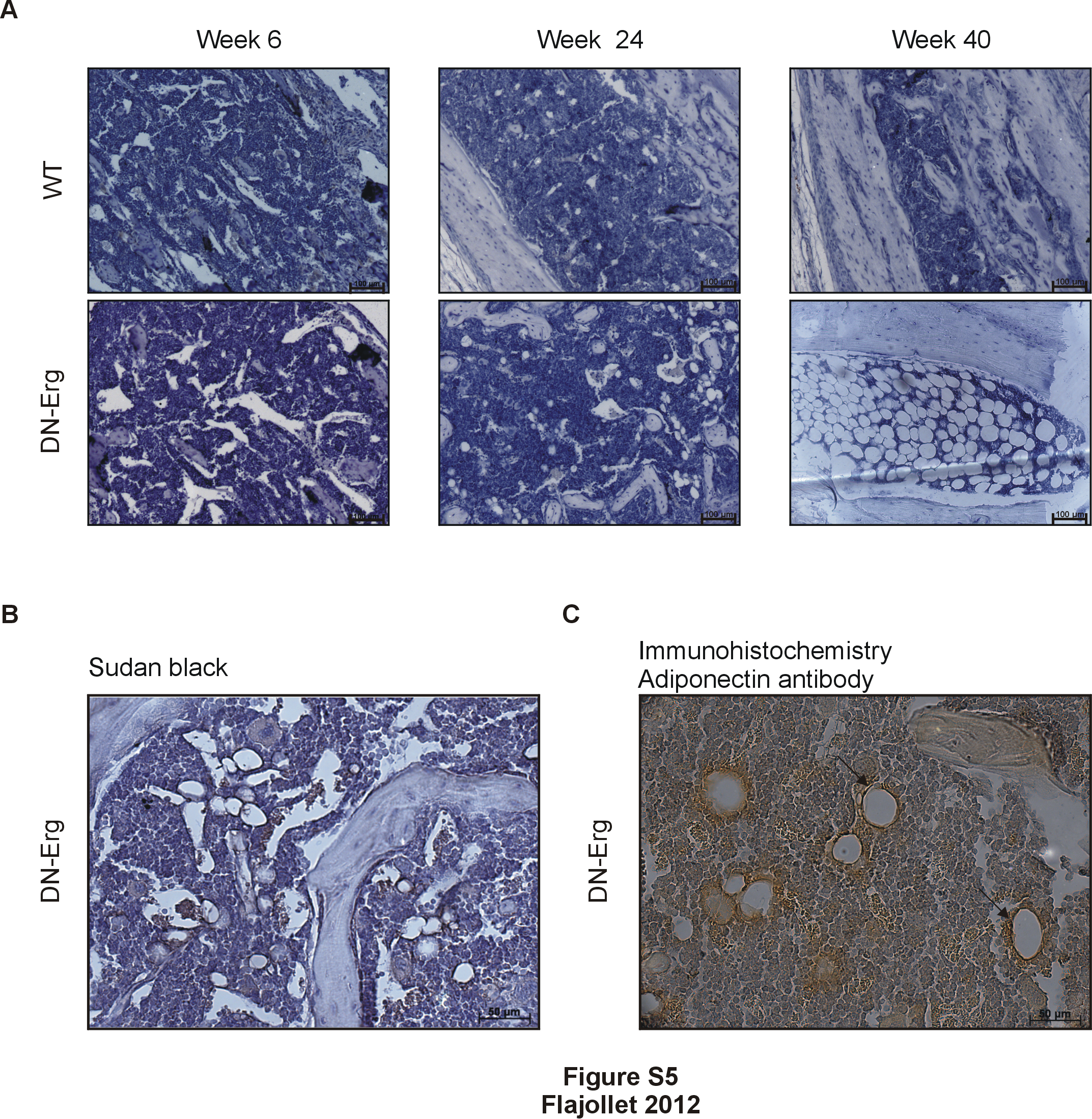

Supplement: Figure S5 — Histological analysis of bone marrow and Adiponectin detection in femur sections of DN-Erg. A. Cytological examination of bone marrow in femur sections of 6, 24 and 40 week-old wt and DN-Erg mice. Scale bar: 100 µm. B. Lipid staining with Sudan black B (Merck) was used to evaluate lipid droplets within adipocytes according to Soldani et al. [61] on section from femur of 40 week-old DN-Erg mice. Scale bar: 50 µm. C. Adiponectin immunocytochemical staining in bone marrow of 40 week-old DN-Erg mice femur. Scale bar: 50 µm. (TIF) [file pone.0048656.s005.tif]
